# Inequitable and Ineffective: Exclusion of Mental Health from the Post-2015 Development Agenda

**DOI:** 10.1371/journal.pmed.1001846

**Published:** 2015-06-30

**Authors:** Alexander C. Tsai, Mark Tomlinson

**Affiliations:** 1 Center for Global Health, Massachusetts General Hospital, Boston, Massachusetts, United States of America; 2 Harvard Center for Population and Development Studies, Cambridge, Massachusetts, United States of America; 3 Mbarara University of Science and Technology, Mbarara, Uganda; 4 Department of Psychology, Stellenbosch University, Stellenbosch, South Africa

## Abstract

Alex Tsai and Mark Tomlinson argue for a place for mental health on the post-2015 development agenda.

The Millennium Development Goals (MDGs), adopted at the turn of the century, represented a milestone in global development by committing United Nations member states to eradicating extreme poverty and achieving specific targets over the subsequent decade and a half. At this time the world's attention is increasingly focused on the post-2015 development agenda, which will be unveiled in September of this year in the form of Sustainable Development Goals (SDGs). Given that mental health and other non-communicable diseases were conspicuously omitted from the MDGs, and have only been weakly mentioned in draft SDG targets, in this essay we argue for a place for mental health on the post-2015 development agenda. Its continued exclusion will not only contribute to the failure of the SDGs given the centrality of mental health in most aspects of human development and well-being but also formalize our collective failure to care for the most vulnerable among us.

## Impact of Omitting Mental Health from the MDGs

The eight MDGs were broadly focused on extreme poverty but also supported improvements in education, public health, and biodiversity—with three health-focused MDGs, related to child mortality; maternal health; and HIV, malaria, and tuberculosis. Although mental disorders [1] and other noncommunicable diseases [2] are substantial contributors to the global burden of disease, they were not formally acknowledged. This omission was a critical oversight, given that interventions to improve mental health likely play an important role in spurring progress on the health-related MDGs [3], including child health [4], maternal health [5], and HIV [6].

Few countries will ultimately achieve the MDG targets for reducing child mortality and improving maternal health [7]. Overall, however, it appears that the MDG platform has been successful in building consensus around, and mobilizing funding for, particular development agendas. Global attention to the issues of newborn survival and maternal health, for example, was facilitated in part by the policy windows created by MDGs 4 and 5 [8,9]. However, the progress that has been made on improving child survival has not been matched by an equivalent focus on ensuring that those that survive also thrive. This oversight has prompted agencies such as the World Health Organization to call for a focus on early child development in the SDGs [10]. Mental health, like early child development, is a cross cutting phenomenon that has the potential to bring together health ministries and diverse actors in a synergistic way that we argue will be essential to achieving the SDGs.

In contrast to the issues of newborn survival and maternal health, the conspicuous exclusion of mental health from the MDGs is thought to have played a significant role in explaining its failure to achieve prominence on the global agenda [11]. Not unrelatedly, most countries in Africa and southeastern Asia devote less than 1% of their health budgets to mental health services [12]. These amounts are supplemented by a paltry amount of development assistance for health, of which less than 1% is earmarked specifically for the care of persons with mental disorders [13].

Fortunately, new initiatives have begun to redress this imbalance of attention, including the *PLOS Medicine* series on Packages of Care [14], Global Mental Health Practice [15], and Integrating Mental Health [16], as well as the 2007 and 2011 *Lancet* series on global mental health [17,18]. Concomitantly, recent initiatives from Grand Challenges Canada, the United Kingdom Department for International Development, and the United States National Institute of Mental Health have begun to redress the imbalance of funding [19–21]. And finally, as indicated in a recent blog posting, World Bank Group President Jim Yong Kim and World Health Organization Director-General Margaret Chan will be cohosting a major event on mental health in the spring of 2016 [22].

## Why Mental Health Should Be Central to the SDGs

The SDG Open Working Group has described poverty eradication as the greatest challenge facing the world. Fittingly, “End poverty in all its forms everywhere” has been designated goal 1 of the proposed SDGs. At this point, there are now 17 proposed goals, of which only one is directly related to health (goal 3, “Ensure healthy lives and promote well-being for all at all ages”), and 169 proposed targets, of which only one is directly related to mental health (target 3.4, “By 2030 reduce by one-third premature mortality from noncommunicable diseases through prevention and treatment, and promote mental health and well-being”). With some projections pegging the cumulative global impact of mental disorders at $16 trillion over the next two decades [23], the exclusion of mental disorders in the SDGs while attempting to eradicate poverty would likely render the SDGs, in Richard Horton’s caustic characterization, as something akin to “fairy tales, dressed in the bureaucratese of intergovernmental narcissism” [24]. The SDGs would then become yet another example of a broad promise in global health left unfulfilled [25].

The importance of recognizing mental health in the SDGs should be obvious. Mental disorders represent some of the most common and disabling sources of human suffering [1], and unmet needs for mental health treatment are pervasive, particularly in resource-limited settings [12,26]. A declared commitment to persons with mental disorders would be an initial step toward supporting those who are among the world’s most marginalized and vulnerable [27], consistent with the “leave no one behind” principle espoused in the UN High-Level Panel report on the post-2015 development agenda [28]. And finally, mental health is directly linked to seven of the proposed SDGs—either as an outcome or as a determinant—namely, those related to poverty, hunger, gender equality, sanitation, employment, inequality, and inclusivity. Mental health is so closely woven into the “triple helix” of sustainable development (economic, social, and environmental) that the continued underdiagnosis and undertreatment of mental disorders makes it highly unlikely that a truly shared and global prosperity will ever be sustainably achieved.

## A More Explicit Commitment to Mental Health

As we count down to September 2015, we see a critical opportunity for the UN General Assembly to formally reaffirm what was recognized in the Political Declaration of the High-Level Meeting of the General Assembly on the Prevention and Control of Non-communicable Diseases [29]: that the continued global burden of mental disorders undermines sustainable human development throughout the world and threatens the achievement of internationally agreed-upon development goals. Our colleagues have already suggested the inclusion of additional targets that would highlight the role of mental health in sustainable human development: avoiding premature deaths (including those from suicide) [30] and achieving parity in access to mental and physical health services [31]. These proposed revisions are substantive and consequential, because the SDGs are likely to have a critical influence on global health and development assistance for the next decade and a half. For a sector that already suffers from underfunding [12] and inattention [11], then, the stakes are exceptionally high.

In summary, mental disorders are common worldwide, can be extremely disabling, and pose grave threats to sustainable human development. Their burden is typically borne by the poorest and most excluded of society, further underscoring the need to “leave no one behind.” The current inequities in mental health service investments, both between and within countries, are unacceptable. Without due attention and formal commitment to the global burden of mental disorders, we fear that the post-2015 goal of ensuring healthy lives and promoting well-being for all will continue to elude us.

## Abbreviations

MDG: Millennium Development Goal
SDG: Sustainable Development Goal
